# Health differentials in the older population of England: An empirical comparison of the materialist, lifestyle and psychosocial hypotheses

**DOI:** 10.1186/1471-2458-11-390

**Published:** 2011-05-25

**Authors:** George B Ploubidis, Bianca L DeStavola, Emily Grundy

**Affiliations:** 1 Department of Population Studies, Faculty of Epidemiology and Population Health, London School of Hygiene and Tropical Medicine, Keppel Street, London WC1E 7HT, UK; 2 Department of Medical Statistics, Faculty of Epidemiology and Population Health, London School of Hygiene and Tropical Medicine, Keppel Street, London WC1E 7HT, UK

## Abstract

**Background:**

In developed countries with old age structures most deaths occur at older ages and older people account for the majority of those in poor health, which suggests a particular need to investigate health inequalities in the older population.

**Methods:**

We empirically compared the materialist, psychosocial and lifestyle/behavioural theoretical mechanisms of explanation for socio-economic variation in health using data from two waves of the English Longitudinal Study of Ageing (ELSA), a nationally representative multi-purpose sample of the population aged 50 and over living in England. Three dimensions of health were examined: somatic health, depression and well-being.

**Results:**

The materialist and lifestyle/behavioural paths had the most prominent mediating role in the association between socio-economic position and health in the older population, whereas the psychosocial pathway was less influential and exerted most of its influence on depression and well-being, with part of its effect being due to the availability of material resources.

**Conclusions:**

From a policy perspective there is therefore an indication that population interventions to reduce health differentials and thus improve the overall health of the older population should focus on material circumstances and population based interventions to promote healthy lifestyles.

## Background

The 20th century witnessed significant improvements in health in most countries including substantial increases in survival to older ages and large reductions in late age mortality. However, substantial inequalities or disparities in the health of different socio-economic groups remain[1-3]. In developed countries with old age structures most deaths occur at older ages and older people account for the majority of those in poor health, which suggests a particular need to investigate health inequalities in the older population [4]. Early work on health inequalities tended to focus on younger age groups, particularly middle aged men. Socioeconomic disparities were thought to be small in early adulthood and later old age and increasingly large during the period between early adulthood and early old age [5], with the declining strength of health inequalities in later life being at least partly attributed to selective mortality. With the availability of new data sources, a growing body of research on older people has demonstrated the persistence of health inequalities at older ages [6,7]. Disentangling the mechanisms underlying health inequalities in the older population is crucial for the development of appropriate policies to alleviate such inequalities and therefore improve population health.

Within the social causation framework and the wider and more extensive theoretical and empirical literature on health inequalities, theories of explanation of the relation between Socio-Economic Position (SEP) and health essentially focus on three mechanisms: [8]. The first is a neo- materialist one; suggesting that those with higher incomes are able to purchase better food, better housing, live in safer environments and have better access to health care[9,10]. According to Lynch [11] "the neo-material interpretation of health inequalities is an explicit recognition that political-economic processes generate income inequality, influence individual economic resources, and also impact community resources such as schooling, health care, social welfare, and working conditions". The second emphasises behavioural or "lifestyle" factors, such as smoking, diet, alcohol consumption and appropriate use of health care, which may also vary with cognitive skill and access to information [12]. The third places more emphasis on psychosocial factors such as empowerment, relative social status and social integration, including exposure to stressful events that may result from low status and low autonomy in important arenas of life, such as work [13]. Health inequalities are thus viewed as the result of perceptions of relative SEP that produce negative emotions which are translated into poorer health through stress[14,15].

In the present study we undertake formal - model based - estimation of the cross sectional and prospective indirect (mediating) effects of SEP on later-life health within a generalised structural equation modelling framework using three dimensions of health. This allows us to compare the relative contribution of each of the three pathways to later-life health inequalities. Such comparisons would not be possible using standard multiple regression models because the latter would estimate only the reciprocally adjusted effects of the variables representing SEP as well as the material, lifestyle and psychosocial pathways, therefore neglecting the indirect perspective, which is crucial for a meaningful comparison of the three mechanisms [16]. However, despite our model being informed by well established theory, the approach we have adopted depends on the model being correctly specified.

The major aim of the present study is to investigate the extent of health inequalities in the older population of England and to empirically estimate the relative contribution to these of the materialist, lifestyle/behavioural and psychosocial pathways, in a first attempt to understand the mechanisms underlying health inequalities. To this explanatory framework we have added a path that captures the association between material resources and psychosocial factors since, as suggested by Wilkinson, [17] we hypothesised that the latter are at least partly contingent upon the availability of the former.

## Methods

### Data source and study sample

We use data from the second (2004) and fourth (2008) waves of the English Longitudinal Study of Ageing (ELSA), a nationally representative multi-purpose sample of the population aged 50 and over living in England. The ELSA sample was drawn from households that responded to the 1998, 1999 or 2001 rounds of the Health Survey for England (HSE), a stratified random sample of all households in England. Response rates to these HSE rounds were 69%, 70% and 67% respectively [18]. A total of 19,924 individuals in households which responded to the HSE would have been aged 50 years by 2002 and so were eligible for inclusion in ELSA. Of these, 11,392 (66%) became ELSA respondents (core participants - see Table 1 for further description of the sample). A comparison of the socio-demographic characteristics of this sample with national census data indicated that the ELSA sample remained representative of the non institutionalised population [18]. The second wave of ELSA included 8,780 core participants and of those 5410 participated in the fourth wave in 2008. We analysed a partially incomplete dataset (N = 8,248), in which participants were included if they had at least one non missing observation in the variables included in the model. As can be seen in Table 1 and in the Appendix, in both waves missing data mostly occurred in the observer measured health indicators, whereas 4242 participants had complete information on all variables included in the model. The sample does not include institutional residents. Although the proportion in institutions is very low in those aged 50-74, particularly among those aged 85 and over the proportion is higher. This means that the most seriously disabled older old are underrepresented in the sample.

**Table 1:** Distribution of SEP indicators, demographic characteristics and other covariates used in the analysis

	*f*	*%*
**Gender**		
**Male**	3949	45.0
**Female**	4831	55.0
**missing**	0	0

**Net Financial Wealth**		
**Wealth 1st fifth (highest)**	1713	19.5
**Wealth 2nd fifth**	1736	19.8
**Wealth 3d fifth**	1727	19.7
**Wealth 4th fifth**	1751	19.9
**Wealth 5th fifth (lowest)**	1724	19.6
**missing**	129	1.5

**Social Class**		
**Managerial and professional**	2415	27.5
**Intermediate**	1533	17.5
**Small employees and own account workers**	859	9.8
**Lower supervisory and technical workers**	968	11.0
**Workers in semi-routine occupations**	2736	31.2
**missing**	269	3.1

**Net Household Income**		
**Income 1st fifth (highest)**	1643	18.7
**Income 2nd fifth**	1697	19.3
**Income 3d fifth**	1739	19.8
**Income 4th fifth**	1785	20.3
**Income 5th fifth (lowest)**	1916	21.8
**missing**	0	0

**Marital Status**		
**Married**	4816	54.9
**Others**	3963	45.1
**missing**	1	0.01

**Education**		
**Degree/Higher education**	2093	23.8
**A level**	575	6.5
**O level/CSE grade**	1870	21.3
**Foreign/other**	764	8.7
**No qualifications**	3468	39.5
**missing**	10	0.1

**Housing Tenure**		
**Owners**	7168	81.6
**Others**	1595	18.2
**missing**	17	0.2

**Ethnicity**		
**White**	8574	97.7
**Not white**	202	2.3
**Missing**	4	0

**Current situation**		
**Retired**	4728	53.8
**Not retired**	4802	55.5
**missing**	74	0.7
**Cognitive Ability (N = 8687)**	**Mean**	**Std Deviation**
**Age (N = 8687)**	-0.042	0.836
	66.50	9.63

Considering that unbiased estimates of pathways, and their components, cannot be obtained without properly addressing the implications of incompleteness we employed the Full Information Maximum Likelihood method which is naturally incorporated into structural equation models. In this full likelihood context model parameters and standard errors are estimated directly from the available data and the selection mechanism is ignorable under the Missing at Random (MAR) assumption [19,20]. The basic goal of FIML missing data handling is to identify the population parameter values that are most likely to have produced a particular sample of data and the discrepancy between the data and the estimated parameters is quantified by the likelihood. In this context the MAR assumption implies that all systematic selection effects depend on variables which are included in the model. In our analyses all potential causes of missingness in the ELSA such as demographic characteristics, SEP indicators, cognitive ability and health status were included in the analysis.

### Measures and variables

#### Exposures - Socioeconomic Position

**Figure 1:**
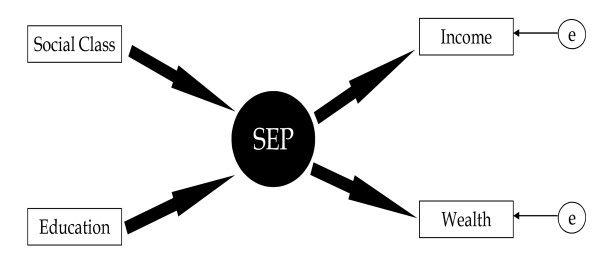
**SEP measurement model**.

We employed several indicators of socio-economic position to derive latent SEP. These were the occupation based National Statistics socio-economic classification (NS-SEC), educational level, net household income and net financial wealth (excluding housing wealth), as suggested by Galobardes[21]. We used the five category version of NS-SEC which allocates people to managerial and professional; intermediate; small employees and own account workers; lower supervisory and technical workers; and workers in semi-routine occupations. Allocation was based on own most recent (or current where applicable) occupation; participants without any information on occupation were excluded from the analysis. Similarly, five educational status groups were derived. The first group comprised participants with a degree or equivalent qualification, the second participants with GCE A level or equivalent qualifications (exams normally taken around age 18), the third respondents with O levels, CSE qualifications or equivalent (exams taken at age 16), the fourth those with foreign qualifications and the fifth those without a formal educational qualification. Finally, net (non housing) financial wealth and equivalised household income were recoded to quintiles. We distinguish between causal-formative indicators such as education and occupational social class, which affect the SEP latent variable, and effect-reflective indicators such as equivalised household income and net financial wealth, which are determined (reflected) by the SEP latent variable [22] (see Figure 1). The four indicators were recoded such that high scores on the SEP latent index represent participants with high SEP.

### Mediators - Measures of the three explanatory pathways

#### Material resources

We employed several indicators to derive a latent summary variable that represents the accumulation of material resources: Housing tenure (recoded to a binary variable distinguishing owners and non-owners), problems with the accommodation (shortage of space, pollution, street noise, cold, etc) as an indicator of neighbourhood affluence; the number of durables owned recoded as an ordinal variable to capture four levels of ownership of durables and to avoid potential problems with skewness; private health insurance as a proxy for access to better health services' and car ownership (see Additional file 1). This latent index represents the accumulation and quality of material resources, with high values indicating participants that own their house, have private health insurance, live in a relatively affluent neighbourhood, own durables such as a television, personal computer, C.D player, microwave oven etc, and own at least one car.

#### Psychosocial path

To define the psychosocial latent dimension we used the four items that represented the control dimension of the CASP-19 questionnaire [23] and two items from the autonomy dimension. We supplemented the CASP items with two additional items present in the ELSA dataset that enquired about perceptions of control at home. High values on this latent index are indicative of participants who perceive their lives as being under their control (see Additional file 1). We employed perceived control as a proxy for the psychosocial path since no information on self esteem or perceived stress is available in the ELSA. It has been shown that the perception of control is an important determinant of coping in the face of hardships, including threats to health [24], as well as a key concept in the explanation of health inequalities [25,26]. When negative events happen the stress that people experience and the resulting health burden due to the accumulation of allostatic load [27] depends on perceived control [27,28] which has also been shown to be associated with stress[29].

#### Health related behaviour-lifestyle

We employed indicators of smoking status (never/past/current smoker) and frequency of exercise in the past month to define latent health related lifestyle. The second wave ELSA dataset does not contain specific questions on dietary habits, but we used the waist hip ratio - appropriately coded for men and women- as a proxy, assuming that participants within the recommended limits make healthier dietary choices (see Additional file 1). This latent index represents the overall health related lifestyle of the participants. High scorers were those who moderately exercise two - three times per week, have never smoked and have a waist-hip ratio within the recommended limits. We chose not to include frequency of alcohol use in the lifestyle latent index, due to the well documented non linear association between alcohol use and various health outcomes [30]. However, we performed a sensitivity analysis including frequency of alcohol use in the measurement model and the correlation between the two lifestyle latent indices (with and without alcohol use) was 0.963, indicating that the exclusion of alcohol did not have any substantial effect on the lifestyle latent index.

### Outcomes - Measures of health

#### Somatic Health

To define this dimension we used the Latent Index of Somatic Health (LISH), which we derived with the procedure proposed by Ploubidis & Grundy [31] in surveys where both self and observer measured health indicators are available. Three observer measured (grip strength; a measure of respiratory function -Forced Vital Capacity - FVC; and chair rise speed) and three self reported health indicators (self rated health, presence of long standing illness, and the presence of one or more functional limitations) were combined with the latent health dimension (LISH) representing somatic health (see Appendix). High scorers on the LISH (optimal somatic health) have satisfactory grip strength, good respiratory functioning, they performed well on the chair rise stands, have no functional limitations neither a chronic illness and perceive their health as very good or excellent.

#### Mental health

As a measure of depression, ELSA includes the eight item version of the CES-D which was developed by Radloff [32] for use in community surveys of adults. We estimated an appropriate measurement model for the eight binary items and used the depression latent continuum in further analysis, as we were interested in variation across the whole range of population mental health, rather than focusing on a particular risk group. High values on the depression latent variable indicate the presence of depressive symptomatology. The ELSA includes Diener's Satisfaction with Life Scale [33] as a measure of subjective well-being. High values on the latent variable indicate participants that report high levels of life satisfaction.

#### Confounders

Age, gender, marital status, ethnicity and a summary measure of cognitive ability at Wave 2 (baseline for this study) were included in the predictive model as they were thought to be important confounders of the relationships between SEP, the three mediators and health.

### Statistical Modelling

**Figure 2:**
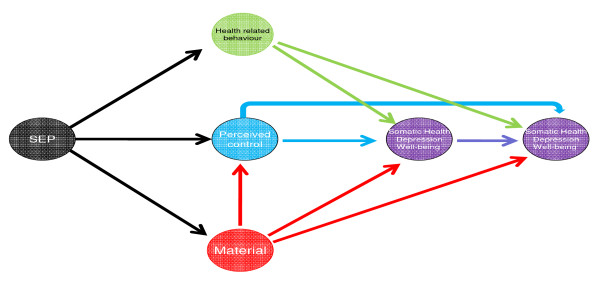
**Conceptual path diagram of the hypothesised structural model**.

Figure 2 shows the conceptual framework that informed our analyses. SEP, material and psychosocial resources and health related lifestyle are latent dimensions which together are assumed to influence health. The latter was defined according to three dimensions - somatic health, depression and well-being. None of these dimensions can be measured directly, i.e. they are latent and are indicated by circles as it is conventional in structural equation modelling. They can nevertheless be identified via appropriate indicators as described below. For simplicity the figure does not separate the three health dimensions, however these are separately identified and allowed to correlate with each other. The specification of each of the latent dimensions described above was at first carried out separately using unidimensional Confirmatory Factor Analytic (CFA) models. The part of the CFA models where ordinal or binary indicators are linked with the continuous latent variables is a normal ogive item response model, similar to the graded responses model [34]. The latent variables represent continuous variables that underlie observed "coarsened" responses such as binary or ordinal responses. The associations between the latent constructs and the manifest indicators are modelled with a 2 parameter probit regression. In this instance, factor loadings represent the strength of the association between the indicator and latent health, whereas the thresholds represent the level of the latent construct that needs to be reached for a particular response in a categorical or ordinal health indicator to be endorsed. The part of the CFA models where continuous indicators are linked with continuous latent factors is a traditional confirmatory factor analytic model with linear regressions between observed and latent variables. In this instance only factor loadings are estimated, and as in the binary/ordinal case they capture the association between the indicators and the latent constructs. Latent trait scores derived from the CFAs were calculated for all predictors, mediators and outcomes in the model. Latent trait scores can theoretically range from -∞ (minus infinity) to +∞ (infinity), but in practice the range is usually from -3 to 3. All latent variable measurement models were estimated with the Weighted Least Squares, Mean and Variance adjusted (WLSMV) estimator in Mplus 5.21 [18]. In the second stage of the analysis the estimated latent trait scores were entered in the path analytic model shown in Diagram 2, which was estimated in order to jointly estimate the direct and indirect associations of SEP with the three health outcomes in both ELSA waves. All reported model parameters are standardized so that their relative sizes can be compared. Estimation was carried out with maximum likelihood, with the "complex" command of the Mplus 5.21 software [18] which accommodates complex sampling designs such as the design of the ELSA. Model fit was assessed with the Comparative Fit Index (CFI), the Tucker Lewis Index (TLI) and the Root Mean Square Error of Approximation (RMSEA). We note that for the CFI and TLI values > 0.90 are indicative of acceptable fit, and values > 0.95 indicative of good fit, whereas for the RMSEA values < 0.08 are indicative of acceptable and values < 0.06 of good fit[35].

## Results

**Figure 3:**
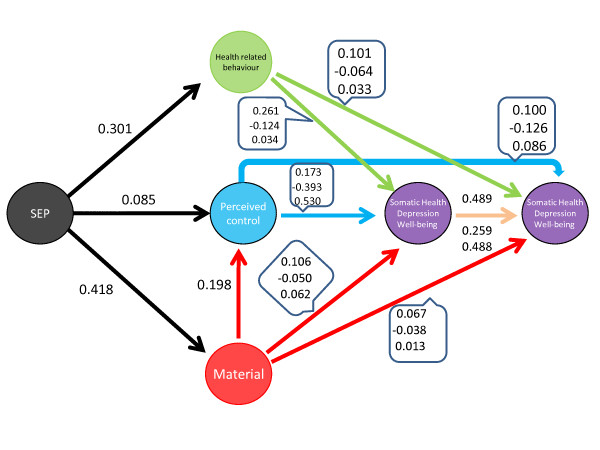
**Path diagram with standardised parameters of the estimated model**.

In Table 2 we present the fit criteria for the CFA measurement models for SEP, the three mediators and the three outcomes. All measurement models had an acceptable fit to the data. In Tables 3 and 4 as well as Figure 3 we present the standardised parameters derived from the path analytic model. We note that parameters in both tables are derived from the same model, but they are presented in two separate tables for clarity.

**Table 2:** Descriptive criteria of model fit

	*CFI*	*TLI*	*RMSEA*
***SEP***	*0.914*	*0.922*	*0.064*
***Material resources***	*0.974*	*0.959*	*0.056*
***Psychosocial ***- ***Perceived control***	*0.951*	*0.948*	*0.064*
***Lifestyle***	*0.967*	*0.942*	*0.027*
***Somatic health***	*0.998*	*0.999*	*0.034*
***Depression***	*0.961*	*0.968*	*0.051*
***Well-being***	*0.992*	*0.994*	*0.062*
***Cognitive ability***	*0.994*	*0.995*	*0.044*
***Path analysis***	0.967	0.903	0.069

**Table 3:** Path analysis standardised parameters - Cross sectional associations

		*Wave 2 Outcomes*			*Mediators*		
		***Somatic Health***	***Depression***	***Well-being***	***Material***	***Psychosocial***	***Lifestyle***
**Gender (Female)**	*Direct*	**-0.203**	-0.013	**0.089**	**-0.104**	0.004	**-0.048**
**Age**	Direct	**-0.368**	**-0.040**	**0.143**	**-0.251**	0.024	**-0.088**
**Marital status (Not Married)**	Direct	**-0.044**	**0.129**	**-0.188**	**-0.240**	**0.047**	**-0.060**
**Ethnicity (Non white)**	Direct	**-0.018**	**-0.053**	0.005	**-0.022**	**-0.029**	-0.019
**Retirement**	Direct	**-0.075**	**0.058**	-0.003	**-0.018**	**-0.079**	**-0.059**
**Cognitive ability**	Direct	**0.029**	**-0.028**	**0.077**	**0.129**	**0.148**	**0.117**
**Material**	Direct	**0.106**	**-0.050**	**0.062**		**0.198**	
**Psychosocial**	Direct	**0.173**	**-0.393**	**0.530**			
**Lifestyle**	Direct	**0.261**	**-0.124**	**0.034**			
**SEP**	Direct				**0.418**	**0.085**	**0.301**
	Indirect via Material	**0.044**	**-0.021**	**0.026**			
	Indirect via Psychosocial	**0.015**	**-0.033**	**0.045**			
	Indirect via Lifestyle	**0.079**	**-0.037**	**0.010**			
	Indirect via Material & Psychosocial	**0.014**	**-0.033**	**0.044**			
	Total	**0.152**	**-0.124**	**0.125**			

*Highlighted parameters are significant

**Table 4:** Path analysis standardised parameters - Prospective associations

		*Wave 4 Outcomes*		
		***Somatic Health***	***Depression***	***Well-being***
**Somatic Health 2002**	*Direct*	**0.489**	**-0.214**	**0.044**
**Depression 2002**	*Direct*	**-0.099**	**0.259**	**-0.098**
**Well -being 2002**	*Direct*	**0.098**	**-0.209**	**0.488**
**Gender (Female)**	*Direct*	**0.075**	**-0.032**	**-0.047**
**Age**	Direct	**-0.102**	**0.043**	**-0.033**
**Marital status (Married)**	Direct	0.012	**0.024**	**-0.030**
**Ethnicity (Non white)**	*Direct*	0.005	-0.018	-0.021
**Retirement**	*Direct*	**-0.019**	0.008	-0.002
**Cognitive ability**	*Direct*	**0.055**	**-0.028**	**-0.027**
**Material**	Direct	**0.067**	**-0.038**	0.013
**Psychosocial**	Direct	**0.100**	**-0.126**	**0.086**
**Lifestyle**	Direct	**0.101**	**-0.064**	**0.033**
**SEP**	Direct			
	Indirect via Material	**0.027**	**-0.015**	0.005
	Indirect via Psychosocial	**0.008**	**-0.010**	**0.007**
	Indirect via Lifestyle	**0.029**	**-0.019**	**0.010**
	Indirect via Material & Psychosocial	**0.008**	**-0.010**	**0.007**
	Indirect via all Wave 2 variables***	**0.096**	**-0.088**	**0.077**
	Total	**0.168**	**-0.142**	**0.106**

*Highlighted parameters are significant

**NA - Not Applicable

*** Outcomes (Somatic health 2002, Depression 2002, Wellbeing 2002) and mediators (material, lifestyle, psychosocial)

### Cross sectional Associations - ELSA Wave 2 (2004)

#### Association between SEP, material resources, perceived control and health related lifestyle

Latent SEP appears to strongly influence latent material resources and latent health related lifestyle, once the influence of gender, age, marital status, cognitive ability, employment status and ethnicity is accounted for. The estimated (standardized) regression coefficients show that one standard deviation increase in SEP score is expected to lead to an increase of *β *= 0.418 p < 0.001 in latent material resources, and an increase of *β *= 0.301, p < 0.001) in the latent lifestyle score. There seems instead to be a weaker association of latent SEP with the psychosocial path (i.e. perceived control), with an estimated standardised regression coefficient *β *= 0.085, p < 0.05. There was also a positive association between the material resources latent index and perceived control, *β *= 0.198, p < 0.001, suggesting that the social patterning of the psychosocial path is at least partly mediated by the accumulation of material resources.

#### Association between material resources, perceived control, health related lifestyle and the three health outcomes

The latent variables which represent the accumulation of material resources and latent perceived control were positively associated with somatic health. The standardised coefficients show that one standard deviation increase in latent material resources is expected to lead to an increase of *β *= 0.106, p < 0.001 in somatic health, whereas an increase in one standard deviation in latent perceived control is expected to result in an increase of, *β *= 0.173, p < 0.001 in somatic health. Similarly latent health related lifestyle was positively associated with somatic health, with one standard deviation increase in latent health related lifestyle leading to a *β *= 0.261, p < 0.001 increase in latent somatic health. All three latent mediators were negatively associated with depression. One standard deviation increase in latent material resources is expected to lead to a *β *= -0.050, p < 0.01 decrease in depression. Likewise one standard deviation increase in latent perceived control is expected to lead to a *β *= -0.393, p < 0.001 decrease in depression. Similarly, one standard deviation increase in latent health related lifestyle will lead to a *β *= -0.124, p < 0.001 decrease in depression. We observed a positive association between the three latent mediators and well-being. One standard deviation increase in latent material resources is expected to result in *β *= 0.062 p < 0.01 increase in well - being. Similarly, one standard deviation increase in latent perceived control (psychosocial path) will result in *β *= 0.530, p < 0.001, increase in well-being. Finally, one standard deviation increase in latent health related lifestyle is expected to lead to *β *= 0.034, p < 0.05, increase in well-being.

#### Comparison between the three explanatory paths

We observed a positive indirect effect of SEP on somatic health via material resources *β_indirect _*= 0.044, p < 0.001, as well as a positive indirect effect via the lifestyle path *β_indirect _*= 0.079, p < 0.001. The indirect effect via perceived control (psychosocial path) was relatively small *β_indirect _*= 0.015, p < 0.05 but the indirect effect of the perceived control (psychosocial path) conditioned on material resources was also significant *β_indirect _*= 0.014, p < 0.05. With respect to depression, the lifestyle path had the strongest mediation effect *β_indirect _*= -0.037, p < 0.001, followed by the psychosocial path *β_indirect _*= -0.033, p < 0.001, whereas the material path had also a substantial mediation effect on depression *β_indirect _*= -0.021, p < 0.001. Furthermore, the indirect - conditioned on material resources- effect via perceived control (psychosocial path), was also significant *β_indirect _*= -0.033, p < 0.001. Most of the effect of SEP on well-being was mediated via the psychosocial path *β_indirect _*= 0.045, p < 0.001. Mediation via the material path was also substantive *β_indirect _*= 0.026, p < 0.001, followed in magnitude by the mediation effect of the lifestyle path *β_indirect _*= 0.010, p < 0.01. The conditioned on material resources mediation effect of the psychosocial path had also a substantive effect on well-being, *β_indirect _*= 0.044, p < 0.05.

### Prospective Associations - ELSA Wave 2 (2004) and ELSA Wave 4 (2008)

#### Association between material resources, perceived control, health related lifestyle and the three health outcomes

The latent variables which represent the accumulation of material resources latent perceived control and latent lifestyle appear to influence somatic health, depression and well-being prospectively, once the effects of gender, age, marital status, cognitive ability, employment status, ethnicity as well as somatic health, depression and well- being at baseline are accounted for. Both material resources and perceived control were positively associated with Wave 4 somatic health. The standardised coefficients show that one standard deviation increase in latent material resources is expected to lead to an increase of *β *= 0.067, p < 0.001 in somatic health, whereas an increase in one standard deviation in latent perceived control is expected to result in an increase of, *β *= 0.100, p < 0.001 in somatic health. Similarly latent health related lifestyle was positively associated with somatic health, with one standard deviation increase in latent health related lifestyle leading to a *β *= 0.101, p < 0.001 increase in Wave 4 latent somatic health. All three latent mediators were negatively associated with Wave 4 depression. One standard deviation increase in latent material resources is expected to lead after four years to a *β *= -0.038, p < 0.01 decrease in depression. Likewise one standard deviation increase in latent perceived control is expected to lead to a *β *= -0.126, p < 0.001 decrease in depression. Similarly, one standard deviation increase in latent health related lifestyle will lead to a *β *= -0.064, p < 0.001 decrease in depression. We observed a positive association between two latent mediators and well-being assessed at Wave 4. One standard deviation increase in latent perceived control (psychosocial path) at baseline will result in *β *= 0.086, p < 0.001, increase in Wave 4 well-being, whereas one standard deviation increase in latent health related lifestyle at baseline is expected to lead to *β *= 0.033, p < 0.05, increase in Wave 4 well-being. We did not observe a significant association between the accumulation of material resources at baseline and Wave 4 well-being.

#### Comparison between the three explanatory paths

We observed a positive indirect effect of SEP on Wave 4 somatic health via material resources *β_indirect _*= 0.027, p < 0.001, as well as a positive indirect effect via the lifestyle path *β_indirect _*= 0.029, p < 0.001. The indirect effect via perceived control (psychosocial path) was relatively small *β_indirect _*= 0.008, p < 0.05 but the indirect effect of the perceived control (psychosocial path) conditioned on material resources was also significant *β_indirect _*= 0.008, p < 0.05. With respect to depression measured at Wave 4, the lifestyle path had the strongest mediation effect *β_indirect _*= -0.019, p < 0.001, followed by the material path *β_indirect _*= -0.015, p < 0.001, whereas the psychosocial path had also a substantive mediation effect on depression *β_indirect _*= -0.010, p < 0.001. Furthermore, the indirect - conditioned on material resources- effect via perceived control (psychosocial path), was also significant *β_indirect _*= -0.010, p < 0.001. Most of the effect of baseline SEP on well-being at Wave 4 was mediated via the lifestyle path *β_indirect _*= 0.010, p < 0.01, followed in magnitude by the mediation effect of the psychosocial path *β_indirect _*= 0.007, p < 0.01. The conditioned on material resources mediation effect of the psychosocial path had also a substantive effect on well-being, *β_indirect _*= 0.007, p < 0.05.

## Discussion

The major aim of the present study was to decompose the effect of later -life SEP on health by empirically comparing three theory driven explanatory pathways using prospective data from the ELSA. Insights on the underlying mechanism of later life health differentials in England have the potential to provide guidance for targeted population interventions as well as health policy and planning to reduce inequalities and promote healthy ageing. We found that the accumulation of material resources had the strongest association with SEP, a finding in accordance with the literature [36,37]. The psychosocial path as represented by perceived control, was the least socially patterned path, confirming previous findings on the association between SEP indicators and perceived control, but also stress as well as cortisol and allostatic load levels[25,26,38,39]. Furthermore, we confirmed previous findings of health inequalities in the older population of England. There was evidence for a social gradient in somatic health, depression and well-being, with low SEP being associated with worse somatic health, depression and lack of well-being [40,41].

Our findings support the neo-materialist and lifestyle hypotheses, since the material and lifestyle paths had the most prominent mediating role in the association between SEP and later -life health, both cross sectionally and prospectively. Furthermore, the observed positive association between material resources and perceived control confirmed our hypothesis that differences in SEP have detrimental psychosocial consequences such as reduced control and self esteem, but these are partly conditioned on the lack of material resources. Our results therefore suggest that at least in the older population, the interpretation of links between SEP and health must begin with the material and health related lifestyle causes of inequalities. We note the effect of the material path on the three health outcomes remained strong even in a model where the added effect of its mediation via the psychosocial path (represented here by perceived control) was not estimated. The lifestyle path was also influential, although health related lifestyle appears to be less socially patterned compared to access to material resources. The psychosocial pathway exerted most of its influence on depression and well-being, a finding that provides further evidence for the social stress hypothesis [15,42]. Taking into account the limitations of observational studies with respect to formally establishing causal associations, our results shed some light on the underlying mechanism of later life health inequalities in England but further research is needed in order to inform population interventions to reduce health differentials and thus improve overall health. We recognise however, that the unconditional to SEP effects of the three mediators on health may not be symmetrical with the effect of their removal, [43] and further research is needed to clarify this issue and its implications to policy decision making.

As with any study, this one has several limitations. First, the selection of variables we employed to represent each explanatory pathway was limited by the secondary nature of our analysis. This limitation is mostly relevant for the psychosocial path since important indicators such as self esteem and perceived stress were not included in the model. However, it has been shown that the perception of control is highly correlated with self esteem and perceived stress [44-46] as well as cortisol and allostatic load[27-29] and therefore can be thought of as an adequate proxy of the psychosocial path. Furthermore, we tried to overcome this limitation by specifying latent summary variables - latent indices- for the three paths. Since our main interest was in the empirical comparison between the three explanatory paths we focused on these latent indices and not on individual indicators or risk factors. We believe that the summary latent indices are an accurate representation of the explanatory paths, especially in situations where important indicators are unmeasured, since one of the properties of latent variable models is their ability to capture unobserved heterogeneity. We hypothesize that the latent indices capture most of the unobserved heterogeneity which is caused by not observed (unmeasured) indicators. Taking into account that for each explanatory path its indicators are expected to correlate, we would not expect the ordering of individuals on the latent indices to change with the addition of unmeasured indicators. This was empirically supported by further sensitivity analysis where direct effects of SEP on the three health outcomes were added (results available from corresponding author). All direct effects were negligible, suggesting that the three paths were captured adequately by the latent summary indices, since according to social theory direct effects between SEP and health are implausible. However, further research is needed with a larger selection of more refined indicators will be available to establish the relative strength of the three paths on later life health.

Another potential limitation is the possibility of confounding on the mediators (the three latent indices) of our model, which we believe is mostly relevant for the lifestyle and psychosocial paths, since if important confounders had been missed, our estimates of direct and indirect effects would be biased[47]. With respect to the lifestyle path, previous evidence suggests that smoking and obesity -reflected in the waist hip ratio- are partly influenced by genetic predisposition [48,49]. We note that the lifestyle latent index captures the common variance between exercise which is a non biologically driven lifestyle choice. The lifestyle latent index thus excludes - to a large extent - any genetic component and the ordering of individuals is largely based on environmentally driven health related lifestyle choices, a property that leads us to conclude that our results would have not been altered substantively if genetic confounders were included in the model. Furthermore, lifestyle choices as well as perceived control which was used here to represent the psychosocial path, may also be influenced by personality traits which have not been measured in the ELSA, but evidence shows that they only partly explain the association between SEP and health [50,51]. Therefore we believe that the lack of personality data may be relevant only for the psychosocial path. Since this was the least influential path on the three health outcomes, we believe that the substantive interpretation of our findings would not have been altered if personality traits were included in the model.

Furthermore, with the exception of somatic health all other variables in our model were self reported. Therefore, response bias may have influenced our results especially with respect to depression and well-being. However, we have no evidence of differential self report bias in the variables that we used as mediators in our model. The implication of this is that - if at all - the effect of the three mediators on depression and well-being was equally biased and therefore the comparison of the three explanatory paths was relatively unaffected. Our analysis was carried out using partially incomplete data. Missing data mostly occurred in the observer measured health indicators in both waves. We estimated several models for sensitivity purposes excluding the observer measured health indicators from the analysis thus increasing the proportion of complete cases in the analysis sample, as well as models with complete data (results available from corresponding author). The results of all these models were similar with the one we present here, suggesting that the inclusion of missing data in our analysis did not bias our results.

Finally, the four SEP indicators were entered in the model simultaneously without a causal specification for their associations. This may have led to an underestimation of the total effect of education, and to a lesser extent occupational social class, since the effect of education on health may operate through a pathway where higher education leads to higher occupational attainment and income, which, in turn, increases the chances of better health[52]. The life course framework offers considerable opportunity to explore these causal pathways. Establishing whether the social patterning of population health occurs at different time periods while recognising the temporal sequence of the associations between SEP indicators will shed further light the life course effect of SEP on later-life health. We note that the effects reported here capture individual level SEP variation, but the extent of health inequalities is also the product of a complex mix of structural - macro level- determinants such as country-level and region-specific background and historical factors that are expected to directly influence health. Our approach may have underestimated the total magnitude of health inequalities, but not the relative contribution of the three paths on the individual level which was our major aim. The inclusion of such measures is beyond the scope of the present study, but we aim to expand the current framework and employ both individual and macro level information within a multilevel modelling approach.

## Conclusions

Our findings support the neo-materialist and lifestyle hypotheses, since the material and lifestyle paths had the most prominent mediating role in the association between SEP and later -life health, both cross sectionally and prospectively. Our results therefore suggest that at least in the older population, the interpretation of links between SEP and health must begin with the material and health related lifestyle causes of inequalities.

## Competing interests

The authors declare that they have no competing interests.

## Authors' contributions

GP conceived the study, performed the statistical analysis and drafted the manuscript, BS participated in the study design and commented on the analytic strategy and early drafts of the manuscript, EG participated in the study design and commented on the analytic strategy and early drafts of the manuscript. All authors read and approved the final manuscript.

## Pre-publication history

The pre-publication history for this paper can be accessed here:

http://www.biomedcentral.com/1471-2458/11/390/prepub

## Supplementary Material

Additional file 1 **Frequency distribution of all indicators of the mediators and outcomes in the structural model**. Frequency distribution of all indicators of the mediators (material resources, perceived control and health related lifestyle) and outcomes (somatic health, depression and well being) in the structural model. Click here for file
