# Supplementary material for: Health differentials in the older population of England: An empirical comparison of the materialist, lifestyle and psychosocial hypotheses
Source: BMC Public Health. 2011 May 25;11:390. doi: 10.1186/1471-2458-11-390 (PMC3128018; doi:10.1186/1471-2458-11-390)
Supplement: Additional file 1 — Frequency distribution of all indicators of the mediators and outcomes in the structural model. Frequency distribution of all indicators of the mediators (material resources, perceived control and health related lifestyle) and outcomes (somatic health, depression and well being) in the structural model. [file 1471-2458-11-390-S1.DOC]

**Frequency distribution of all indicators of the mediators and outcomes in the structural model**

Distribution of material path indicators

| **Housing tenure** | | | **Problems in accommodation** | |  | **Durables** | | |
| --- | --- | --- | --- | --- | --- | --- | --- | --- |
|  | *f* | *%* |  | *f* | *%* |  | *f* | *%* |
| Others | 1595 | 18.2 | *Yes* | *2557* | *29.1* | <1 | 342 | 3.9 |
| Owners | 7168 | 81.6 | *No* | *6186* | *70.5* | 2-4 | 1978 | 22.5 |
| Missing | 17 | 0.2 | *Missing* | *37* | *0.4* | 5-8 | 4170 | 47.5 |
| Total | 8780 | 100 | *Total* | *8780* | *100* | >8 | 2267 | 25.8 |
|  |  |  |  |  |  | *Missing* | *23* | *0.3* |
|  |  |  |  |  |  | *Total* | *8780* | *100* |
| **Private health insurance** | | | **Car ownership** | | |  |  |  |
|  | f | % |  | *f* | *%* |  |  |  |
| No | 7510 | 85.5 | *No* | *1989* | *22.7* |  |  |  |
| Yes | 1267 | 14.4 | *Yes* | *6791* | *77.3* |  |  |  |
| Total | 8777 | 100 | Missing | 0 | 0 |  |  |  |
| Missing | 3 | 0.1 | Total | 8780 | 100 |  |  |  |
| Total | 8780 | 100 |  |  |  |  |  |  |

**Distribution of lifestyle path indicators**

| **Smoking** | | | | **Moderate excersise** | | |
| --- | --- | --- | --- | --- | --- | --- |
|  | *f* | *%* | |  | *f* | *%* |
| Current smoker | 1341 | 15.3 | | Hardly | 1019 | 11.6 |
| In the past | 238 | 2.7 | | 2-4 times per month | 2374 | 27 |
| Never | 7139 | 81.3 | | 2 (or more) times a week | 5292 | 60.3 |
| Missing | 62 | 0.7 | | Missing | 95 | 1.1 |
| Total | 8780 | 100 | | Total | 8780 | 100 |
|  | | |  | | | |
| **Waist - hip ratio** |  |  | |  |  |  |
|  | *f* | *%* | |  |  |  |
| Outside limits | 6780 | 77.2 | |  |  |  |
| Within limits | 614 | 7.0 | |  |  |  |
| Missing | 1386 | 15.8 | |  |  |  |
| Total | 8780 | 100 | |  |  |  |
|  |  |  | |  |  |  |

**Distribution of psychosocial path (perceived control) indicators**

| **A*ge prevents* *from doing things*** | | | ***Out of their control*** | | | ***Plan for the future*** | | |  |
| --- | --- | --- | --- | --- | --- | --- | --- | --- | --- |
|  | *f* | *%* |  | *f* | *%* |  | *f* | *%* |  |
| Often | 713 | *8.1* | *Often* | *441* | *5* | *Never* | *700* | *8* |  |
| Sometimes | 2143 | *24.4* | *Sometimes* | *1474* | *16.8* | *Not often* | *946* | *10.8* |  |
| Not often | 2*273* | *25.9* | *Not often* | *2370* | *27* | *Sometimes* | *2271* | *25.9* |  |
| Never | 2470 | *28.1* | *Never* | *3160* | *36* | *Often* | *3476* | *39.6* |  |
| Missing | 1181 | *13.5* | *Missing* | *1335* | *15.2* | *Missing* | *1387* | *15.8* |  |
| Total | 8780 | *100* | *Total* | *8780* | *100* | *Total* | *8780* | *100* |  |
| **Left out of things** | | | ***Things they want to do*** | | | **Responsibilities *prevent*** | | |  |
|  | *f* | *%* |  | *f* | *%* |  | *f* | *%* |  |
| Often | 339 | *3.9* | *Never* | *381* | *4.3* | *Often* | *343* | *3.9* |  |
| Sometimes | 1432 | *16.3* | *Not often* | *648* | *7.4* | *Sometimes* | *1784* | *20.3* |  |
| Not often | 2213 | *25.2* | *Sometimes* | *2196* | *25* | *Not often* | *1921* | *21.9* |  |
| Never | 3495 | *39.8* | *Often* | *4329* | *49.3* | *Never* | *3424* | *39* |  |
| Missing | 1301 | *14.8* | *Missing* | *1226* | *14* | *Missing* | *1308* | *14.9* |  |
| Total | 8780 | *100* | *Total* | *8780* | *100* | *Total* | *8780* | *100* |  |
| **P*lease themselves what they do*** | | | **C*ontrol over what*  *happens at home*** | | | **S*et by factors beyond their* *control*** | | |  |
|  | *f* | *%* |  | *f* | *%* |  | *f* | *%* |  |
| Never | 331 | *3.8* | *Strongly disagree* | *75* | *0.9* | *Strongly agree* | *1342* | *15.3* |  |
| Not often | 508 | *5.8* | *Moderately disagree* | *90* | *1* | *Moderately agree* | *2642* | *30.1* |  |
| Sometimes | 2268 | *25.8* | *Slightly disagree* | *140* | *1.6* | *Slightly agree* | *1917* | *21.8* |  |
| Often | 4465 | *50.9* | *Slightly agree* | *540* | *6.2* | *Slightly disagree* | *557* | *6.3* |  |
| Missing | 1208 | *13.8* | *Moderately agree* | *3136* | *35.7* | *Moderately disagree* | *668* | *7.6* |  |
| Total | 8780 | *100* | *Strongly agree* | *3690* | *42* | *Strongly disagree* | *467* | *5.3* |  |
|  |  |  | *Missing* | *1109* | *12.6* | *Missing* | *1187* | *13.5* |  |
|  |  |  | *Total* | *8780* | *100* | *Total* | *8780* | *100* |  |

**Distribution of somatic health indicators**

| **Self reported indicators** | | *f* | *%* |
| --- | --- | --- | --- |
| ***Functional limitations*** | |  |  |
| FL problems reported | | 5258 | 59.9 |
| No limitations | | 3522 | 40.1 |
| Missing | | 0 | 0 |
| ***Self-rated general health*** | |  |  |
| Poor | | 679 | 7.7 |
| Fair | | 1794 | 20.4 |
| Good | | 2755 | 31.4 |
| Very good | | 2391 | 27.2 |
| Excellent | | 1063 | 12.1 |
| missing | | 98 | 1.1 |
| ***Long standing (chronic) illness*** | |  |  |
| Yes | | 5062 | 57.7 |
| No | | 3713 | 42.3 |
| missing | | 5 | 0.1 |
| **Observer measured indicators** | **Mean** | | **Standard deviation** |
| Grip strength (kg) (N = 7508) 29.18 | | | 11.51 |
| Chair rise (seconds) (N= 6368) | 11.71 | | 4.32 |
| Lung function (liters) (N = 7025) | 3.15 | | 1.10 |

**Distribution of depression indicators**

| **D*epressed much of the time*** | | | **W*as an effort*** | | | ***Restless during the* *past week*** | | | |
| --- | --- | --- | --- | --- | --- | --- | --- | --- | --- |
|  | *f* | *%* |  | *f* | *%* |  | *f* | *%* |  |
| *No* | *7198* | *82* | *No* | *6661* | *75.9* | *No* | *4965* | *56.5* |  |
| *Yes* | *1419* | *16.2* | *Yes* | *1956* | *22.3* | *Yes* | *3647* | *41.5* |  |
| *Missing* | *163* | *1.9* | *Missing* | *163* | *1.9* | *Missing* | *168* | *1.9* |  |
| *Total* | *8780* | *100* | *Total* | *8780* | *100* | *Total* | *8780* | *100* |  |
| ***Happy much of the time*** | | | **F*elt lonely*** | | | ***Enjoyed life*** | | | |
|  | *f* | *%* |  | *f* | *%* |  | *f* | *%* |  |
| *Yes* | *7696* | *87.7* | *No* | *7396* | *84.2* | *Yes* | *7751* | *88.3* |  |
| *No* | *901* | *10.3* | *Yes* | *1219* | *13.9* | *No* | *848* | *9.7* |  |
| *Missing* | *183* | *2.1* | *Missing* | *165* | *1.9* | *Missing* | *181* | *2.1* |  |
| *Total* | *8780* | *100* | *Total* | *8780* | *100* | *Total* | *8780* | *100* |  |
| ***Felt sad*** | | |  | ***Could not get going*** | |  |  |  |  |
|  | *f* | *%* |  | *f* | *%* |  |  |  |  |
| *No* | *6763* | *77* | *No* | *6770* | *77.1* |  |  |  |  |
| *Yes* | *1848* | *21* | *Yes* | *1838* | *20.9* |  |  |  |  |
| *Missing* | *169* | *1.9* | *Missing* | *172* | *2* |  |  |  |  |
| *Total* | *8780* | *100* | *Total* | *8780* | *100* |  |  |  |  |

**Distribution of well-being indicators**

| ***Life is close to ideal*** | | | | **C*onditions of life are excellent*** | | | **S*atisfied with their life*** | | |
| --- | --- | --- | --- | --- | --- | --- | --- | --- | --- |
|  | f | | % |  | *f* | *%* |  | *f* | *%* |
| Strongly agree | 179 | | *2* | *Strongly agree* | *188* | *2.1* | *Strongly agree* | *103* | *1.2* |
| Agree | 438 | | *5* | *Agree* | *459* | *5.2* | *Agree* | *316* | *3.6* |
| Slightly agree | 382 | | *4.4* | *Slightly agree* | *492* | *5.6* | *Slightly agree* | *352* | *4* |
| Neither agree nor disagree | *1165* | | *13.3* | *Neither agree nor disagree* | *792* | *9* | *Neither agree nor disagree* | *517* | *5.9* |
| Slightly disagree | 1476 | | *16.8* | *Slightly disagree* | *1540* | *17.5* | *Slightly disagree* | *1101* | *12.5* |
| Disagree | 3302 | | *37.6* | *Disagree* | *3130* | *35.6* | *Disagree* | *3887* | *44.3* |
| Strongly disagree | 643 | | *7.3* | *Strongly disagree* | *926* | *10.5* | *Strongly disagree* | *1378* | *15.7* |
| Missing | 1195 | | *13.6* | *Missing* | *1253* | *14.3* | *Missing* | *1126* | *12.8* |
| Total | 8780 | | *100* | *Total* | *8780* | *100* | *Total* | *8780* | *100* |
| **G*ot the important things they want in life*** | | | | **L*ive their life again, would change nothing*** | | |  |  |  |
|  | | f | *%* |  | *f* | *%* |  |  |  |
| Strongly agree | | 81 | *0.9* | *Strongly agree* | *400* | *5.2* |  |  |  |
| Agree | | 264 | *3* | *Agree* | *834* | *10.9* |  |  |  |
| Slightly agree | | 271 | *3.1* | *Slightly agree* | *784* | *10.2* |  |  |  |
| Neither agree nor disagree | | *415* | *4.7* | *Neither agree nor disagree* | *647* | *8.4* |  |  |  |
| Slightly disagree | | 1062 | *12.1* | *Slightly disagree* | *1372* | *17.9* |  |  |  |
| Disagree | | 3785 | *43.1* | *Disagree* | *2495* | *32.6* |  |  |  |
| Strongly disagree | | 1773 | *20.2* | *Strongly disagree* | *1129* | *14.7* |  |  |  |
| Missing | | *1129* | *12.9* | *Missing* | *1119* |  |  |  |  |
| Total | | 8780 | *100* | *Total* | *8780* |  |  |  |  |

**Distribution of somatic health indicators – ELSA Wave 4, 2008**

| **Self reported indicators** | | *f* | *%* |
| --- | --- | --- | --- |
| ***Functional limitations*** | |  |  |
| FL problems reported | | 4196 | 47.8 |
| No limitations | | 1954 | 22.3 |
| Missing | | 2630 | 30 |
| ***Self-rated general health*** | |  |  |
| Poor | | 424 | 4.8 |
| Fair | | 1260 | 14.4 |
| Good | | 2015 | 22.9 |
| Very good | | 1696 | 19.3 |
| Excellent | | 624 | 7.1 |
| missing | | 2761 | 31.4 |
| ***Long standing (chronic) illness*** | |  |  |
| Yes | | 3493 | 39.8 |
| No | | 2656 | 30.3 |
| missing | | 2631 | 30 |
| **Observer measured indicators** | **Mean** | | **Standard deviation** |
| Grip strength (kg) (N = 5221) 27.20 | | | 12.13 |
| Chair rise (seconds) (N= 4364) | 9.29 | | 6.29 |
| Lung function (liters) (N = 3953) | 3.10 | | 1.06 |

**Distribution of depression indicators – ELSA Wave 4, 2008**

| **D*epressed much of the time*** | | | **W*as an effort*** | | | ***Restless during the* *past week*** | | | |
| --- | --- | --- | --- | --- | --- | --- | --- | --- | --- |
|  | *f* | *%* |  | *f* | *%* |  | *f* | *%* |  |
| *No* | 5142 | 58.6 | *No* | 4838 | 55.1 | *No* | 5395 | 61.4 |  |
| *Yes* | 847 | 9.6 | *Yes* | 1147 | 13.1 | *Yes* | 578 | 6.6 |  |
| *Missing* | 2791 | 31.8 | *Missing* | 5985 | 68.2 | *Missing* | 2807 | 32.0 |  |
| *Total* | 8780 | 100 | *Total* | 8780 | 100 | *Total* | 8780 | 100 |  |
| ***Happy much of the time*** | | | **F*elt lonely*** | | | ***Enjoyed life*** | | | |
|  | *f* | *%* |  | *f* | *%* |  | *f* | *%* |  |
| *Yes* | 5395 | 61.4 | *No* | 5216 | 59.4 | *Yes* | 5440 | 62 |  |
| *No* | 578 | 6.6 | *Yes* | 775 | 8.8 | *No* | 535 | 6.1 |  |
| *Missing* | 2807 | 32 | *Missing* | 2789 | 31.8 | *Missing* | 2805 | 31.9 |  |
| *Total* | 8780 | 100 | *Total* | 8780 | 100 | *Total* | 8780 | 100 |  |
| ***Felt sad*** | | |  | ***Could not get going*** | |  |  |  |  |
|  | *f* | *%* |  | *f* | *%* |  |  |  |  |
| *No* | 4819 | 54.9 | *No* | 4794 | 54.6 |  |  |  |  |
| *Yes* | 1169 | 13.3 | *Yes* | 1194 | 13.6 |  |  |  |  |
| *Missing* | 2792 | 31.8 | *Missing* | 2792 | 31.8 |  |  |  |  |
| *Total* | 8780 | 100 | *Total* | 8780 | 100 |  |  |  |  |

**Distribution of well-being indicators – ELSA Wave 4, 2008**

| ***Life is close to ideal*** | | | | **C*onditions of life are excellent*** | | | **S*atisfied with their life*** | | |
| --- | --- | --- | --- | --- | --- | --- | --- | --- | --- |
|  | *f* | | % |  | *f* | *%* |  | *f* | *%* |
| Strongly agree | 137 | | 1.6 | *Strongly agree* | 135 | 1.5 | *Strongly agree* | 94 | 1.1 |
| Agree | 340 | | 3.9 | *Agree* | 354 | 4 | *Agree* | 221 | 2.5 |
| Slightly agree | 311 | | 3.5 | *Slightly agree* | 404 | 4.6 | *Slightly agree* | 299 | 3.4 |
| Neither agree nor disagree | 784 | | 8.9 | *Neither agree nor disagree* | 608 | 6.9 | *Neither agree nor disagree* | 423 | 4.8 |
| Slightly disagree | 1198 | | 13.6 | *Slightly disagree* | 1319 | 15 | *Slightly disagree* | 924 | 10.5 |
| Disagree | 2121 | | 24.2 | *Disagree* | 1910 | 21.8 | *Disagree* | 2612 | 29.7 |
| Strongly disagree | 333 | | 3.8 | *Strongly disagree* | 464 | 5.3 | *Strongly disagree* | 716 | 8.2 |
| Missing | 3556 | | 40.5 | *Missing* | 3586 | 40.8 | *Missing* | 3491 | 39.8 |
| Total | 8780 | | 100 | *Total* | 8780 | 100 | *Total* | 8780 | 100 |
| **G*ot the important things they want in life*** | | | | **L*ive their life again, would change nothing*** | | |  |  |  |
|  | | *f* | *%* |  | *f* | *%* |  |  |  |
| Strongly agree | | 52 | 0.6 | *Strongly agree* | 276 | 3.1 |  |  |  |
| Agree | | 183 | 2.1 | *Agree* | 560 | 6.4 |  |  |  |
| Slightly agree | | 215 | 2.4 | *Slightly agree* | 646 | 7.4 |  |  |  |
| Neither agree nor disagree | | 361 | 4.1 | *Neither agree nor disagree* | 497 | 5.7 |  |  |  |
| Slightly disagree | | 841 | 9.6 | *Slightly disagree* | 1069 | 12.2 |  |  |  |
| Disagree | | 2678 | 30.5 | *Disagree* | 1682 | 19.2 |  |  |  |
| Strongly disagree | | 945 | 10.8 | *Strongly disagree* | 562 | 6.4 |  |  |  |
| Missing | | 3505 | 39.9 | *Missing* | 3488 | 39.7 |  |  |  |
| Total | | 8780 | 100 | *Total* | 8780 | 100 |  |  |  |
